# Uniform and Complementary Social Interaction: Distinct Pathways to Solidarity

**DOI:** 10.1371/journal.pone.0129061

**Published:** 2015-06-05

**Authors:** Namkje Koudenburg, Tom Postmes, Ernestine H. Gordijn, Aafke van Mourik Broekman

**Affiliations:** University of Groningen, Groningen, The Netherlands; Lanzhou University, CHINA

## Abstract

We examine how different forms of co-action give rise to feelings of solidarity. We propose that (a) coordinated action elicits a sense of solidarity, and (b) the process through which such solidarity emerges differs for different forms of co-action. We suggest that whether solidarity within groups emerges from uniform action (e.g. synchronizing, as when people speak in unison) or from more complementary forms of action (e.g. alternating, when speaking in turns) has important consequences for the emergent position of individuals within the group. Uniform action relies on commonality, leaving little scope for individuality. In complementary action each individual makes a distinctive contribution to the group, thereby increasing a sense of personal value to the group, which should contribute to the emergence of solidarity. The predictions receive support from five studies, in which we study groups in laboratory and field settings. Results show that both complementary and uniform co-action increase a sense of solidarity compared to control conditions. However, in the complementary action condition, but not in the uniform action (or synchrony) condition, the effect on feelings of solidarity is mediated by a sense of personal value to the group.

## Introduction

Researchers often distinguish between groups and social categories. Group research tends to focus on small dynamic groups with some form of interdependence and social interaction. By contrast, studies of social categories often focus on group members’ perceptions of large social groups that exist by virtue of some shared property such as nationality or ethnicity (e.g., [1]). Although categorical processes appear to be more prevalent in large groups and interactive processes in small groups [2–3] we believe that both sets of processes occur in all groups (small and large) to some extent. In the present paper, our broad aim is to learn more about the operation of interactive and categorical processes in small groups, in order to understand how feelings of solidarity emerge.

Solidarity may emerge from the recognition of similarities between individuals: Uniformity of characteristics or actions fosters both perceptions of entitativity and social categorization (e.g., [4–7]). But solidarity can also emerge through interactions that appear to be much less uniform ([8–10]). Most social interactions tend to consist of sequences of complementary actions: In conversations, for example, people take turns making distinctive contributions. Interestingly however, the same groups that engage in dialogic interaction may, at other occasions, express and develop solidarity through uniform actions such as communal prayer, dance, etc.

Although uniformity and complementarity may both foster a sense of solidarity, we propose that the process is very different because the individual group members play such different roles in the group’s formation. In groups that interact in a uniform fashion, a sense of unity could be derived from the ability to distinguish the own group from its social context, thereby placing the individual in the background, cf. [11–12]. In groups in which members interact in more complementary ways however, the distinctive input of each individual is a fundamental part of the group’s actions, making each individual of personal value to group formation. It is this distinction that is central to the current research.

### Two Pathways to Solidarity

In the Oxford English Dictionary solidarity is defined as “the fact or quality, on the part of communities etc., of being perfectly united or at one in some respect, especially in interests, sympathies, or aspirations”. In sociological and social-psychological theorizing, the concept of solidarity has been used to explain the ways in which communities are tied together (e.g. [13]) or to specify some sort of attachment of belonging to a group [14]. Accordingly, we use the term solidarity here to refer to both the experience that an aggregate of individuals constitutes a social unity (i.e. the entitativity of a group), and the feeling that one is part of this social unity (i.e. the sense of belonging or identification with this group).

A broad range of theories proposes that similarity is a key predictor of solidarity. According to the similarity-attraction hypothesis [15–16] people are more likely to feel attracted to similar others. In group research, self-categorization theory (SCT: [12], [17–18]) proposes that people are most likely to categorize as group members when differences within the group are smaller than differences between groups. According to SCT, individuals tend to perceive themselves in terms of a shared stereotype that defines the ingroup in contrast to relevant outgroups (e.g., [19]).

Postmes et al. [1] argued that this type of group formation echoes some characteristics of Durkheim’s [13] concept of *mechanical solidarity*: A form of solidarity anchored in commonalities or concurrent actions. Durkheim associated mechanical solidarity with groups including indigenous tribes, who used rhythmic co-action to increase and express group unity. Indeed, more recent research has supported the idea that people synchronize their behavior in interactions [20–22] and that such synchronous interaction increases not only group entitativity (the perception of unity of the group as an entity) but also interpersonal liking (the strength of interpersonal relations within the group) and cooperative behavior [5–6], [23–25]. Moreover, synchronous movement has been shown to blur self-other boundaries: Even complete strangers perceived themselves as more similar to each other and showed more conformity to each other after synchronous, rather than a-synchronous stimulation [26–28].

In modern societies however, Durkheim suggested that solidarity is *organic*: here individual complementarity serves as the basis for group formation and the individuality of group members becomes an important consideration in group functioning. Durkheim provides the example of a village composed of different craftsmen. Here, it is the way in which craftsmen complement and build upon each other, rather than the similarity of craftsmen, that provides a sense of solidarity in the village. Complementarity thus refers to the integrated and coordinated actions of individuals who, by virtue of their actions, are quite dissimilar from each other (or to be more precise: distinctive without being antagonistic).

Durkheim’s observations can be related to contemporary research showing that interpersonal interaction is also a major predictor of feelings of entitativity and improved interpersonal relations within the group [3], [8–10], [29–31]. This can be conceptualized as a bottom-up process in which a common sense of identity is *induced* from group members’ individual contributions to the group [32–34]. Further research has shown that also in heterogeneous groups, inductive processes can provide a strong basis for identification [2].

In sum, there are two distinct ways in which solidarity can be achieved. One could be termed deductive (or mechanical): overarching similarities in the group influence group members to experience solidarity. This solidarity can be witnessed in different conceptually related indicators of solidarity including entitativity and social identification. Exactly the same indicators of solidarity are affected by a second pathway, which we termed inductive (or organic): The complementary actions of individual group members creating a successful community.

In the research by Postmes and colleagues, the process of identity formation is manipulated directly to be either inductive or deductive. The idea behind this is that this creates different types of solidarity, which has consequences for, for instance, the way group members deal with heterogeneity within the group (e.g. [2], [35]). The present research builds on these prior studies, zooming in on the process of co-action in groups and its consequences for social solidarity. But rather than manipulating identity formation directly, we merely vary the mode of social interaction between group members: We believe that the way members of the group interact with each other shapes the development of a sense of solidarity.

### Sense of Personal Value to the Group

One of the differences between mechanical and organic processes of group formation lies in the contributions that individual group members make to it. Durkheim already observed that in organic societies there would be more scope for individuality. Indeed, if solidarity is based on member similarity, there is little scope for individuality within the group. Group members should feel mutually replaceable and have little individual value to the group as a whole. For example, the solidarity between soldiers in a platoon is often based upon the principle that all are equal. This is embodied through uniform clothing, as well as synchronous action (e.g., marching, drill exercises). The similarity or replaceability of soldiers in their formation or units could be beneficial for the army’s continuity in combat: The loss of individuals would not endanger the performance of a unit so long as their membership could be refreshed. The army and its units were (and to a large extent are) designed so that the loss of individual lives does not endanger the functioning of the organization. In such situations, feelings of solidarity are presumably less anchored in individual features, and based more on group features (platoon, division, branch, nation).

Conversely, when group formation is organic, the actions of individuals in the group are a direct determinant of the physical manifestation of the group. In a conversation, for instance, the flow of talk can only proceed smoothly if speakers organize their speech production and comprehension so that they take turns, reflect upon the other’s utterances, etc. [36–38]. To function as a coherent social unit, the input of all members in such organic group processes is essential: When one person or subgroup was to leave, the group would change. In other words, coordinating who talks when, and building upon what has been said by other speakers allows members to form a social structure [9–10]. The structure of an organically formed group, for example as it emerges in a conversation, is based on the complementarity of the individual contributions to the group. Previous research suggested that the recognition of one’s distinctive input within the group has positive consequences for personal wellbeing and can enhance a sense of connection [39–41]. Therefore, we expect that in such organic or complementary structures, the sense of personal value to the group will be an important predictor of an emergent sense of solidarity.

### The Present Research

In the present paper we examine whether feelings of solidarity can emerge in the background of group members’ coaction. We propose a model in which coordinated action elicits a sense of solidarity. We measure three aspects of solidarity: First, we examine group members’ perceptions of group *entitativity*, i.e. the extent to which they perceive their group as a social unit. Second, we assess the extent to which group members *identify* with the group. Third, we examine the extent to which group members feel that they *belong* to the group. Although it is clear that these three are closely related, we included them because they are central to different schools of thought in group research. Thus, entitativity is an important construct in interdependence research and refers to perceived unity at the collective level. Identification is an important variable in the social identity tradition, and refers to feelings of attachment to the group as an entity. Belongingness, finally, has been examined in research on ostracism and is linked in that literature to individual needs. Although these three concepts stem from distinct conceptual traditions, we believe they all tap into a sense of solidarity within the group. One could hypothesize that the three should be differentially affected by our manipulations. However, in line with the literature review above, we believe that it would be likely for all three variables to be affected in similar ways by coordinated action.

Additionally, we propose that this sense of solidarity emerges quite differently for complementary and uniform actions, respectively. When group members undertake complementary actions, for instance by taking turns in a conversation, a sense of personal value plays an important role. Here, the group members’ sense of solidarity is founded upon the integration of a unique combination of contributions from individual members. In contrast, when group members undertake uniform actions, such as when talking or singing in synchrony, identification processes are less likely to be influenced by a person’s personal value to the group. Therefore, we expect that in the complementary action condition, but not in the uniform action (synchrony) condition, the emergence of solidarity is mediated by the feeling that one is personally valuable to the group.

Finally, the different ways in which solidarity can emerge may affect group outcomes. For instance, the complementarity of behavior and subsequent experience of personal value to the group could foster divergent thinking. The reasoning behind this is that a complementary action group derives solidarity not from uniformity, but is likely to value group members’ distinctiveness in behavior and thinking. This may become one of the group’s strengths when such increased divergence of thought leads to enhanced creativity compared to group whose solidarity is derived from uniform action.

In this research, we hypothesize that a) both complementary and uniform (synchronous) action can increase solidarity in the form of increased perceptions of group entitativity, and increased identification with, and belonging to the group, b) a sense of personal value mediates the relation between complementary action and feelings of solidarity, but not the relation between uniform action and feelings of solidarity, and c) compared to uniform action, complementary action leads to more divergence in a subsequent idea generation task, promoting creativity in groups.

We tested this model in five studies using different methods. All studies we performed in this line of research are reported in the present paper. Study 1 examines the general distinction between naturally occurring solidarity through uniform action and solidarity through complementary action. Additionally, in Study 1 we develop a measure of sense of personal value to the group and examine whether it distinguishes between groups whose solidarity emerged from either of the two forms of coaction. In Study 2, we manipulate different forms of coordination (synchrony vs. complementarity) in dyads. We examine whether this leads to solidarity and how each of these forms is related to a sense of personal value. In Study 3, we test the same hypotheses in a different context (i.e., a choir) and with triads. Study 4 aims to replicate Study 2 and 3 in again a different context, namely amongst actors. Importantly, in Study 4 we also investigate the consequences of different social structures for group creativity and idea generation. Finally, Study 5 focuses on alternative explanations for the effects, in particular whether the different amount of effort involved in both forms of coordination may confound the effects. In addition, Study 5 examines whether a sense of personal value is only related to solidarity because individuals value themselves, or whether the value of others may also contribute to the emergent sense of solidarity.

## Study 1

In Study 1, we examined whether people would recognize both processes in group settings that naturally occurred in their daily life, and we examined what associations they had with these different settings. We asked participants to remember social experiences from their personal life in which they performed complementary actions or uniform actions. It was hypothesized that both situations promote equal levels of entitativity, identification, and belonging (H1), that a sense of personal value to the group is higher in the complementary action condition than in the uniform action condition (H2), and that this sense of personal value mediates the effect on the indicators of solidarity in the complementary action condition, more so than in the uniform action condition (H3).

### Method

#### Ethics statement

The research was approved by the Ethical Committee Psychology of the University of Groningen. Participants had a minimum age of 16, and were allowed to provide their own informed consent by the Ethical Committee Psychology of the University of Groningen. Written informed consent was obtained on paper (in Studies 2, 3 and 4) or digitally (Studies 1 and 5) by all participants immediately before the research commenced.

### Participants and design

The sample consisted of 199 participants (*M*age = 21.01, *SD* = 6.85, 74% female) who were recruited via the undergraduate participant pool at the University of Groningen (n = 164), or via various online forums (n = 35). Undergraduates participated for partial course credit; the other participants were volunteers. Participants were randomly assigned to the conditions of a study in which coordination (uniform action vs. complementary action) was manipulated by remembering a situation in which they behaved similarly or complementary to others.

### Procedure

Participants filled out an online questionnaire on ‘social situations’. They were asked to think back to a group setting. In the uniform action condition it was stated: “Sometimes group members all perform actions that are roughly similar. Please take your time to think back to a situation in which you did something together with other people, and in which everyone acted more or less similarly.” In the complementary action condition participants read “Sometimes group members all perform different actions. Please take your time to think back of a situation in which you did something together with other people, and in which everyone had a unique input.” Participants were then asked whether they recognized this kind of situation, and to describe such a situation from their own experience. The recalled experiences were coded by a trained coder, who was blind to the conditions of the study. Subsequently, participants were asked to fill out a questionnaire about this experience.

### Dependent variables

The questionnaire assessed participants’ sense of personal value to the group. We developed a measure consisting of three items; “I had an important role in this group”, “I think I was indispensable to this group”, “Without me, this group would not function”, and found this to have adequate reliability, Cronbach’s α = .87. In addition, participants completed a 4-item entitativity scale ([2] e.g., “I feel that the others and I are a unit”, α = .91) and a 14-item social identification scale ([14] e.g., “I feel a bond with this group”, α = .94). Feelings of belonging were measured by 4 items derived from the Need Threat Scale ([42] e.g., “During the task I felt that I belonged with the others” α = .89). As manipulation checks, participants indicated the extent to which they agreed with four items: In this situation “Everyone did something different”, “Every group member had a different input” (*action complementarity*: α = .84), and in this situation “Everyone acted the same”, “All group members had the same input (*action uniformity*: α = .78). All variables were measured on a scale from 1 = strongly disagree, to 7 = strongly agree.

### Results

Seven participants were unable to remember a situation and their data were removed before the analyses (N complementary action condition = 5, N uniform action condition = 2). No outliers (Studentized Residuals > 3) were detected. An analysis of variance (ANOVA) on the manipulation check revealed that group members perceived the situation that they reported to have more action complementarity in the complementary action condition than in the uniform action condition: *M* = 5.21, *SD* = 1.09 and *M* = 3.43, *SD* = 1.51 respectively, *F*(1, 185) = 85.32, *p* < .001, η^2^ = .32. Conversely, group members perceived the situation that they reported to have less action uniformity in the complementary action condition than in the uniform action condition: *M* = 3.14, *SD* = 1.32 and *M* = 4.70, *SD* = 1.32 respectively, *F*(1, 185) = 65.03, *p* < .001, η^2^ = .32.

### Description of situations

In the uniform action condition, participants mentioned behaviors such as playing sports and games (23%), going to a party, including behaviors such as dancing (7%), eating or drinking (13%), and chatting or laughing (12%). In addition, they mentioned situations which were characterized by some form of conformity to the group (14%), e.g. “The first time I went smoking, I smoked because everybody else did”, “During a hazing ritual we all acted similarly (for instance when eating or singing) because we were told to”, “We once went to a shop where we all bought something healthy, just because we did not want to look stupid”.

In the complementary action condition, participants mentioned things that involved organizing an activity or event (34%) including things like “everyone painted a different part of the house”, “We organized a New Year’s Eve party, and everyone had their own task. One organized the drinks; someone else arranged a location, etc.” In addition, participants mentioned making a school- or work assignment (33%), and sports or games that were characterized by a distinct input of each player (7%).

### Dependent variables

As predicted, participants had a stronger sense of personal value in the complementary action condition than in uniform action condition, *F*(1, 190) = 9.83, *p* = .002, η^2^ = .05. In line with the predictions, no differences in perceived entitativity (*F*(1, 190) = 1.49, *ns*), feelings of belonging (*F* < 1, *ns*) and identification (*F* < .1, *ns*) were found. Means are summarized in Table 1; correlations between the different indicators of solidarity are summarized in Table 2.

**Table 1:** Means (SD’s) for the dependent variables in Study 1.

	Uniformity (n = 99)	Complementarity (n = 93)
**Personal Value to Group**	3.45 (1.48)	4.12 (1.45)
**Entitativity**	5.28 (1.23)	5.05 (1.31)
**Belonging**	5.54 (1.13)	5.39 (1.07)
**Identification** *	4.73 (1.18)	4.79 (1.14)

*For identification there were 3 missing values.

**Table 2:** Pearson correlations between the different indicators of solidarity (entitativity, belonging and identification) for each of the studies.

		Belonging	Identification
**Entitativity**	**Study 1**	.80***	.64***
	**Study 2**	.85***	.84***
	**Study 3**	71***	-
	**Study 4**	.74***	.53***
	**Study 5**	.74***	.69***
**Belonging**	**Study 1**		.72***
	**Study 2**		.83***
	**Study 3**		-
	**Study 4**		.37***
	**Study 5**		.67***

Note. Unilevel correlation coefficients are reported.

***p < .001.

### Indirect effect

As expected, we did not find differences between conditions on the indicators of solidarity. However, we predicted that there is a relative difference in the extent to which complementary action (vs. uniform action) yields feelings of solidarity via a sense of personal value to the group. To test this, we estimated the indirect effect of complementary action (vs. uniform action) via personal value on perceived entitativity, identification, and belonging using the bootstrapping procedure developed by Hayes [43]. The effect size of the indirect effect is indicated by K^2^ [44]. The analyses revealed an indirect effect of condition via personal value on identification (*B* = .13, SE = .06, 95% bootstrapped CI [.04; .28], *K* ^2^ = .06), perceived entitativity (*B* = .24, SE = .09, 95% bootstrapped CI [.09; .44], *K* ^2^ = .10), and belonging, (*B* = .21, SE = .08, 95% bootstrapped CI [.08; .39], *K* ^2^ = .11). When modeling this effect, the direct effect of complementary action on perceived entitativity became negative, *B* = -.46, SE = .17, *t* = -2.69, *p* = .01, a suppression effect suggesting that a sense of personal value contributes to why perceptions of entitativity in complementary groups are as high as in uniform action groups. A similar negative direct effect appeared for belonging, after modeling the effect of personal value, *B* = -.36, SE = .15, *t* = -2.41, *p* = .02. No direct effect of condition on identification was found (*t* < 1, *ns*).

### Discussion

Study 1 shows that in recollections of real-life group situations, high complementarity was associated with situations that are descriptively very distinct from high uniformity. Thinking about uniformity evoked a broad range of situations revolving around shared social activities whose main purpose appears to be communal enjoyment (e.g., having fun through socially scripted and symbolic forms of interaction). When participants were asked to recall complementary action, they recalled situations that were much more instrumental and focused on achievement of some common goal (e.g., collaborative work to achieve some desirable outcome). Despite the marked difference between both kinds of activities recalled, they were associated with approximately equal levels of perceived group entitativity, experienced belonging and identification. However, compared to uniform action situations, group members recalling complementary situations experienced a higher sense of personal value, and this predicted their feelings of solidarity.

Although we find Study 1 of descriptive interest and suggestive of the social processes that are central to this paper, we believe that for various reasons (the correlational nature of the data, the inability to control for confounds, the reliance on explicit recollection for tapping into processes that might be of an implicit nature) we cannot draw any firm conclusions. Study 2 therefore experimentally studied the emergence of solidarity “in the background” of a particular dyadic activity that participants were asked to perform. In order to examine whether feelings of solidarity would emerge as a result of the co-action, a control condition was included in Study 2.

## Study 2

### Method

Seventy-six undergraduate students (*M*age = 19.08, *SD* = 1.68, 66 female, 10 male) participated in a study for partial course credit or a single reward of 5 euros. The sample size in each of the following studies was based on a minimum of 20–25 per condition [45]. Because this is dyadic or triadic data, however, individual studies may still be somewhat underpowered if intraclass correlations (ICC) are very high. Previously unacquainted dyads were randomly assigned to one of 3 conditions (control vs. synchrony vs. complementarity). Two dyads knew each other beforehand. Analyzing the data without these two dyads yielded similar results.

### Procedure

Participants entered the lab individually and were seated in separate cubicles after which they were assigned to a partner. After filling out an informed consent form, participants were instructed to read a story through headsets together with their assigned partner. The story was one page long and concerned a man who visited a restaurant. In the synchrony condition, participants were instructed to read the story simultaneously (in sync) with their partner. In the complementarity condition, participants read the sentences of the story in turn. In the control condition, participants read the story and were informed that their partner was reading the story in the next cubicle. It took dyads about 5 minutes to read the whole story. After reading, participants took off their headsets and filled in a questionnaire. Finally, participants were fully debriefed and thanked for their participation.

This research builds on prior research (e.g., [30]) that examines the impact of smoothly coordinated interaction to various control conditions, including a condition in which interactions are disrupted by silences. Accordingly, this first experimental study contained two conditions in which we attempted to disrupt group collaborations by brief delays in auditory feedback. But this disruption manipulation failed: In the turn-taking condition a short delay disrupted interaction in the predicted way, but in the synchronous interaction condition it caused complete breakdown of interaction in several groups. Because this means that delay conditions are no longer equivalent and comparable and because these conditions are not relevant for the current paper, we decided not to report them.

### Dependent variables

Participants’ sense of personal value to the dyad (α = .78), entitativity (α = .93), and belonging (α = .96) were measured as in Study 1. Identification was assessed with three subscales of the Leach et al. identification scale ([14], α = .92): Solidarity (α = .93), satisfaction (α = .90) and homogeneity (α = .88). Because the groups consisted of only two members, the self-stereotyping subscale was deemed less relevant. In addition, because these were newly formed dyads, we thought that questions about the centrality of the group to the individuals’ identity would not make any sense to some of the participants. Therefore, we did not measure these identification subscales. The dependent variables reported in the paper were embedded in a larger questionnaire which contains additional variables (again, see [30]). We have only reported the most central dependent variables here, but the full set of results is available from the first author.

### Results

Two orthogonal Helmert contrasts were specified: ψ1 differentiated between coordinated interaction (synchrony and complementarity) and the control condition. ψ2 differentiated between the synchrony and the complementarity condition. The intra-class correlations (ICC1; [46]) for entitativity (.54), identification (.61), belonging (.80) suggested that multilevel analysis was required. The sense of personal value had a much lower ICC1 (.03), which is consistent with the idea that this is an assessment of distinctiveness made at the individual level. To account for the interdependence of the data, we used Hierarchical Multilevel Analysis. Means are summarized in Table 3.

**Table 3:** Means (SD’s) for the dependent variables in Study 2.

	Control	Synchrony	Complementarity
	(n = 21)	(n = 28)	(n = 27)
**Personal Value to Group**	3.46 (1.53)	3.70 (1.16)	4.27 (1.25)
**Entitativity**	2.55 (1.09)	4.18 (1.14)	4.94 (1.00)
**Belonging**	2.17 (.86)	5.10 (1.07)	5.78 (.71)
**Identification**	2.84 (.89)	4.49 (.91)	4.76 (.89)

### Solidarity

Individual-level perceptions of entitativity, belonging and identification were regressed onto dyad-level contrasts ψ1 and ψ2. The analysis showed that participants who had a coordinated interaction perceived their dyad to be more entitative than participants in the control condition, ψ1: γ = 2.02, *SE* = .30, *t*(36) = 6.67, *p* < .001. In addition, participants in the complementarity condition perceived their dyad to be more entitative than those in the synchrony condition, ψ2: γ = .76, *SE* = .32, *t*(36) = 2.40, *p* = .022.

Similarly, participants who had a coordinated interaction felt more belonging to the group than participants in the control condition, ψ1: γ = 3.28, *SE* = .26, *t*(36) = 12.68, *p* < .001. In addition, participants in the complementarity condition felt that they belonged more to the group than those in the synchrony condition, ψ2: γ = .69, *SE* = .27, *t*(36) = 2.53, *p* = .016.

Finally, participants in the coordinated interaction conditions identified stronger with their dyad than participants in the control condition, ψ1: γ = 1.80, *SE* = .26, *t*(36) = 6.85, *p* < .001. No difference was found between the complementarity and the synchrony condition (ψ2: *t* < 1).

### Personal value to the dyad

A similar analysis showed no significant effect of ψ1 on sense of personal value to the dyad: γ = .52, *SE* = .33, *t*(36) = 1.56, *p* = .13, although mean scores on personal value were somewhat higher in the interaction conditions than in the control condition. In addition, ψ2 did not significantly affect participants’ sense of personal value, γ = .58, *SE* = .35, *t*(36) = 1.63, *p* = .11, but means were in the predicted direction: Participants in the complementarity condition had a somewhat higher sense of personal value than those in the synchrony condition.

### Mediation

We tested two different mediation hypotheses: One for the indirect effect of synchrony (vs. control, dummy D1) through a sense of personal value on the indicators of solidarity; and one testing the same effect for complementarity (vs. control, dummy D2). This was a multilevel mediation: *Condition* was a group level (2) variable, which predicted *sense of personal value to the group* and *entitativity*, *belonging*, *and identification* at the individual level (1). We followed guidelines provided by Preacher, Zyphur, and Zhang [47] for conducting a 2-1-1 multilevel mediation. As predicted, there was no evidence for mediation of the synchrony condition effect, via personal value, on identification (γ = .30, *SE* = .50, *t* < 1, *ns*), nor on entitativity (γ = .30, *SE* = .82, *t* < 1, *ns*), nor on belonging (γ = .25, *SE* = .43, *t* < 1, *ns*). However, two trends suggested indirect effects of complementarity (D2) via personal value on identification, γ = 1.02, *SE* = .68, *t*(36) = 1.49, *p* = .14, 95% CI [-.32; 2.36], and belonging, γ = .88, *SE* = .55, *t*(36) = 1.61, *p* = .11, 95% CI [-.19; 1.96]. No evidence for a mediation of the effect on entitativity was found, γ = 1.04, *SE* = 2.00, *t* < 1, *ns*, 95% CI [-2.88; 4.95]. Although the indirect effects are not statistically significant, the direction of the effects is in line with the hypothesis that in the complementarity condition, but not in the synchrony condition, participants’ sense of personal value to the group would predict their levels of belonging and identification with the group.

### Discussion

Study 2 showed that having a coordinated interaction with a partner increased feelings of solidarity. Participants who read a story together perceived their dyad to be more entitative, felt that they belonged more, and identified more with their dyad than participants in a control condition. In addition, participants who read the story by taking turns with the other member of their dyad reported slightly higher perceptions of entitativity and higher levels of belonging than those who read the story in synchrony. No significant effects of the different ways of coordinating speech on identification were found. The findings regarding entitativity and belonging were unexpected and suggested that, if anything, complementary action led to slightly stronger feelings of solidarity than synchronous action did. Although it is possible that these findings reflect a theoretically meaningful difference, it could also be a side-effect of the methodology we used in this study. For instance, it is possible that reading in turns through headset resembles a telephone conversation, in which turn-taking is the default way of coordinating speech. The experienced familiarity when taking turns in this situation compared to simultaneous speech, may have fostered the development of a sense of solidarity in this condition. Study 3 was designed to test whether this finding would hold in a different context.

Study 2 also revealed that a sense of personal value to the group was related to two of the three indicators of solidarity. Results are thus at least somewhat consistent with the idea that personal value can create a sense of solidarity on a basis quite distinct from the homogeneity beliefs that are often assumed to be central to social identity formation (cf. [35]).

It was hypothesized that participants’ sense of personal value would increase when taking turns, rather than reading in synchrony. The data did not provide significant support for this hypothesis, although the means were in the predicted direction. However, it is quite likely that a dyad is too small for members to negate personal value to the group. Indeed, in order to consider a dyad a “group”, even in the synchrony condition both participants are required: A group of one is not typically considered a group. Study 3 therefore examined groups of three participants to account for this limitation.

Moreover, in Study 2 participants in the experimental conditions were more aware of their partner than in the control condition—who did not see or hear their partners at any time during the experiment. It is therefore possible that participants in the control condition felt that they were actually alone during the experiment, which may have confounded the effects. In the following studies, partners in each condition were therefore able to see and hear their partners throughout the experiment.

## Study 3

Because speaking in synchrony is a more uncommon activity than speaking in turns, we wanted to replicate these findings across several settings. One such context in which synchronous and complementary group activities can be compared is singing. The activity of singing together has often been suggested to increase a sense of togetherness [48–50]. Indeed, many groups, such as sororities, churches, and tribes use singing in their activities, pointing to the symbolic relevance of this group activity. Research on the effect of singing on the experience of togetherness has (to our best knowledge) almost exclusively focused on the act of singing in unison [49]. However, singing often also occurs in more complementary forms where multiple voices can be discerned, for example in part-singing, in duets or in canon. A recent study showed that compared to singing a canon or multiple voice part, singing in unison leads to higher synchronization of cardiac and respiratory patterns between people [51]. Different forms of singing thus affect bodily synchronization between people. However, the psychological consequences of such synchronization are yet to be examined. The present study contrasted singing in unison to complementary singing. We reasoned both would lead to an increased sense of solidarity to the group, but that the role of individuality in the process would be very different.

### Method

Thirty-one singers (*M*age = 40.49, *SD* = 15.89, range 14–65; 6 male, 25 female) participated voluntarily in a field-study advertised to be about “singing together”. Participants were informed about the study via their choirs, their singing teachers, or posters in the music institute and signed up for the study individually. All participants participated voluntarily. After arrival at the music institute, participants consecutively went through all three conditions (control vs. synchrony vs. complementarity) in random order, each time with a different group of two or three singers. Thus, for each round, participants were randomly assigned to groups, which were randomly assigned to one of three conditions.

In each of the conditions participants were asked to sing the song *Use Somebody* (written by the Kings of Leon). Three days before the study, participants received the lyrics and a link to the vocal and piano version of the song (performed by Laura Jansen). In the synchrony condition, members of the group were instructed to sing the song simultaneously, in unison. In the complementarity condition, members were instructed to sing by taking turns on each new line in the song. In the control condition, each group member was instructed to sing the first two verses of the song solo, in presence of the other group members. In each condition, the singers were accompanied by a piano.

After each round, participants filled out a questionnaire assessing their sense of personal value to the group (α = .84), perceptions of entitativity (α = .90), and feelings of belonging (α = .84) similar to previous studies. Because the design of Study 3 required that participants filled out the same questionnaire three times, we reduced the length of the questionnaire by not including a measure of identification. Additionally, participants indicated the extent to which they felt that they had a *voice* in the group, with 5 items: “I had the ability to make my own voice heard”, “I dared to make my own voice heard”, “I could be myself in the group”, “I could be different than others in this group”, “I tried to make my own voice heard”, α = .79. This variable was created to distinguish between participants perceived *scope* for individual action (their voice) and their perceptions of these actions as meaningful contributions to the group as a whole; which would lead to increased sense of personal value to the group.

In order to not make it too apparent to participants that the study was concerned with people’s feelings of solidarity, these questions were embedded in a larger list of filler items about various aspects of the singing, e.g., the perceived aesthetics of the performance, various feelings aroused by the singing, etc. After the third round of questionnaires, participants were fully debriefed and had the opportunity to ask questions.

### Results

Again, two contrasts were specified to differentiate between conditions in which participants were singing together and the control ‘solo’ condition (ψ1), and between the synchrony and the complementarity condition (ψ2). Hierarchical Multilevel Analysis with Cross-classified effect modeling was used to correct for the interdependence of the data. The outcomes were measured at level 1. This level was nested within individuals (each individual participated 3 times), and within groups (each group consisted of three individuals). We found no influence of order (whether it was the first, second, or third round of the experiment). In theory, one could also model the influences of group members in the previous round, on the individual outcomes of the next round. However, to reduce complexity, we did not include these models.

When screening for multilevel outliers, two outliers appeared. Because these participants appeared normal on the other measures, and we preferred not to remove single measurements from our dataset, we decided to test our hypotheses both with and without the outliers. No differences emerged, except for a marginally significant effect of ψ2 on entitativity: β = -.43, *SE* = .26, *t*(86) = -1.67, *p* = .10, Because of the nested structure of our model and the small sample size, we report the data with all cases included. However, two participants could only be included in two of the three conditions; One of them participated in only two of three rounds and the other did not completely fill out one of the questionnaires. Means are summarized in Table 4. The within participant ICC1s for personal value to the group (.66), entitativity (.39), belonging (.04), and voice (.51) indicated that we needed to correct for interdependence of the data on the level of the individual. Within groups, the ICC1s for personal value to the group (.07) and voice (.07) were quite low, but the ICC1s for entitativity (.14) and belonging (.12) indicated that there was variance that could be explained at the group level.

**Table 4:** Means (SD’s) per condition for the dependent variables in Study 3.

	Solo	Synchrony	Complementarity
	(n = 29)	(n = 31)	(n = 31)
**Personal Value to Group**	4.26 (1.37)	3.91 (1.46)	4.38 (1.93)
**Belonging**	4.47 (1.31)	5.04 (1.24)	5.12 (1.22)
**Entitativity**	4.01 (1.37)	4.37 (1.49)	4.10 (1.18)
**Voice**	6.01 (.81)	5.38 (.87)	5.65 (1.07)

#### Solidarity

A regression including both contrasts at the group-level was performed to predict measurement-level entitativity with the group, while correcting for the level of the individual. No between-condition differences were found for perceptions of entitativity, ψ1: *t* < 1, *ns*, and ψ2: *t* < 1, *ns*.

A similar analysis on feelings of belonging showed the predicted effect: Participants who were singing together (either in synchrony or in complementarity) experienced higher feelings of belonging than participants in the control condition ψ1: β = .64, *SE* = .29, *t*(88) = 2.24, *p* = .03. No differences between the synchrony and complementarity condition were found, ψ2: *t* < 1, *ns*.

### Personal value to the group

No effects of ψ1 on sense of personal value to the group were found, *t* < 1, *ns*. However, on ψ2, a marginally significant effect in the predicted direction was found suggesting that participants in the complementarity condition felt they had a higher personal value to the group than those in the synchrony condition,: β = .45, *SE* = .26, *t*(88) = 1.76, *p* = .08.

#### Voice

Participants perceived that they had more voice in the control condition, than in the conditions in which they sang together, ψ1: β = -.47, *SE* = .14, *t*(88) = -3.38, *p* = .001. In addition, a marginally significant effect on ψ2 suggested that participants in the complementarity condition felt that they had more voice than those in the synchrony condition, β = .26, *SE* = .16, *t*(88) = 1.68, *p* = .096.

### Process

We examined whether feelings of belonging and perceptions of entitativity could be predicted by sense of personal value to the group. Because of the complex structure of our model, we decided not to examine mediation, but assess the relations between variables with cross-classified multilevel regressions. These regressions indicated that a sense of personal value predicts both entitativity (θ = .18, *SE* = .09, *t*(89) = 1.96, *p* = .052), and belonging (θ = .28, *SE* = .08, *t*(89) = 3.74, *p* = .001). Voice positively predicts belonging (θ = .31, *SE* = .14, *t*(89) = 2.30, *p* = .024) but does not significantly predict entitativity (θ = -.11, *SE* = .15, *t* < 1, *ns*). Finally, voice was related to a sense of personal value to the group, θ = .87, *SE* = .12, *t*(89) = 6.76, *p* < .001.

### Discussion

Study 3 shows that singing together, compared to singing alone, increases feelings of belonging. Perceptions of entitativity do not change as a result of the way of singing. The data reveal a marginally significant effect suggesting that compared to singing in unison, singing in turns increases a sense of personal value to the group. These feelings are related to a sense of belonging and perceptions of entitativity. Together these results suggest that singing in a complementary fashion can elicit feelings of belonging and entitativity up to a level similar as singing in unison, possibly because of an increased sense of personal value to the group. The effect on personal value to the group is however statistically marginal. Possibly, the effect is obscured by the generally high levels of noise in data that is acquired through real-life interaction (or, in this case, singing together), but it may also be that the effect, in fact, is random. Study 4 therefore aims to replicate this finding in a between subjects design.

Comparable to the results on personal value, Study 3 showed that participants felt that they had more *voice* in the complementarity condition, than in the synchrony condition. The variable voice related to the extent to which people felt that they could make their own voice heard. However, whereas a sense of personal value to the group was related to perceptions of group entitativity, voice appeared to be unrelated to group entitativity. This possibly suggests that feelings of group unity may depend less on being given scope for independent action than on making a recognizable contribution to a group product.

In Study 3, we did not find that singing together increased entitativity compared to a control condition in which participants were singing solo. Because we did not a priori expect the solo condition to increase solidarity or a sense of personal value to the group, we did not define this contrast in our analyses. However, from the means and standard deviations, we can conclude that there are no differences between the sense of personal value to the group in the solo condition and in the complementary condition. Possibly, the experience of singing solo in the presence of others emphasized the relation between singer and ‘audience’, therefore eliciting a sense of entitativity in itself. Supporting this idea, we found that the mean sense of personal value to the group in the solo condition was almost as high as the mean in the complementarity condition, suggesting that participants may have experienced some form of complementarity when singing solo. This was a limitation, because Study 3 now lacked a ‘true’ control condition to which the effects on entitativity could be compared. In Study 4 we therefore included a control condition for which the development of different actor-audience relations would be less likely.

## Study 4

Together, the first three studies suggest that a sense of solidarity can emerge through co-action. The results also show that complementary actions elicit a structure that is qualitatively different from uniform action with regard to the position of the individual. Study 4 focuses on the consequences of these different forms of solidarity for the level of divergence within groups.

### Convergence and Divergence within Groups

In social structures in which similarity is the defining feature of the group, behavior that deviates from the norm is a problem to the internal cohesion of the group. Indeed, research suggests that in such groups, norm deviations are experienced as threats to the distinctiveness of the own group with regard to other groups and therefore often elicit punishment [52–53].

Research has shown that such a search for consensus can lead to a convergent style of thinking, in which group members are likely to concentrate on the proposed viewpoint to the exclusion of other considerations [54–56]. For instance, they are likely to discuss information that is already shared among group members, rather than bring new facts to the table [57].

Whereas members of groups in which solidarity emerges from similarities are likely to think in a convergent manner, groups in which solidarity emerges from complementary action may not function in a similar way. For instance, when members are assigned expert roles, this can lead to more coordinated information sharing, in which members mutually recognize each other’s responsibility for specific domains of information [58]. Similarly, norms that promote individualism, originality or critical thought can decrease sanctions against dissenting group members [33], [59–60]. Taking this a step further, this research suggests that in groups that are based on individual contributions, voicing dissimilar opinions may be less harmful for the group’s social identity. After all, it is not their distinctiveness from other groups that informs members about who they are as a group, but rather the individual coordination amongst members that promotes a sense of solidarity. In line with this reasoning, exposure to minority viewpoints has been shown to elicit more divergent thought [54] and heterogeneous groups have been suggested to be more effective in problem solving than homogeneous groups [61] (but see [62] for a review of different effects of different types of heterogeneity).

Taking this together, Study 4 tests the hypothesis that groups in which solidarity emerges through complementary action are more likely to think in a divergent manner than groups in which solidarity emerges through uniformity. That is, we expect complementary action to increase the generation of both more ideas (fluency) and more original ideas (originality), which are argued to contribute to creativity, problem solving and decision making [54], [63].

### Coordinated Action in Theatre

In Study 4, we employed actors to read out a text in synchrony or in turns. Actors were chosen because both forms of synchronous speech and complementarity (e.g., turn-taking) are naturally occurring in plays as well as in practice sessions. In fact, in ancient Greek tragedies or comedies, synchronous speaking in *unison* is a normal occurrence: It is the mode in which the chorus observes and comments on the action of the actors. Interesting to note is that in Greek drama, the chorus often repeats portions of the text that have also appeared in dialogue. It has been suggested that this “vox populi” affirms the statements made by individuals through the public and renders it truthful (a form of social validation, in other words [64]). A contemporary version of synchronous speech is often incorporated in modern plays, such as musicals or grand operas and this form is a well-rehearsed aspect of actors’ training.

### Method

Ninety-three actors (*M*age = 22, *SD* = 4.61, 57 female, 36 male) participated in groups of three in a field study for a single reward of 5 euros. Groups were randomly assigned to the conditions of a study in which interpersonal coordination was manipulated (synchronous vs. complementarity vs. control) by reading a poem.

Participants were recruited at different professional and amateur theater companies and schools. After filling out the informed consent form participants of all groups were instructed to recite the Dutch translation of the poem *The Raven* by *Edgar Allan Poe*. In the synchrony condition, participants were instructed to recite the poem simultaneously with the other participants, in the same rhythm. In the complementarity condition, they were instructed to recite the sentences of the poem in turn. In the control condition, participants were instructed to recite the poem, independently of each other. To make sure that participants did not synchronize in this condition, they were positioned in such a way that they could not hear each other. Afterwards, they completed a questionnaire assessing their sense of personal value to the group (α = .80), perceptions of entitativity (α = .85), feelings of belonging (α = .80) and identification (α = .92) in the same way as in Study 2.

### Group creativity task

After filling out the questionnaire, all groups received the instructions for a group creativity task. They were asked to write a promotion plan for a theater play of Romeo and Juliet (Shakespeare). Groups were asked to discuss how to handle the promotion, and to write down their plan on an A4-paper. They were given 15 min to complete the task, and during this time the experimenter left the room. The group task was videotaped for later analysis. Finally, participants were fully debriefed.

### Results

As in Study 2, two contrasts were specified: ψ1 differentiated between coordinated interaction (synchrony and complementarity) and no coordinated interaction (control), ψ2 differentiated between the synchrony and the complementarity condition. The ICC1’s for entitativity (.43), identification (.47), belonging (.39) and sense of personal value to the group (.15) suggested that multilevel analysis was needed. One multilevel outlier was removed (Standardized residual on one of the dependent variables > 3). Means are summarized in Table 5.

**Table 5:** Means (SD’s) per condition for the dependent variables in Study 4.

	**Control**	**Synchrony**	**Complementarity**
	**(n = 29)**	**(n = 30)**	**(n = 33)**
**Personal Value to Group**	2.72 (1.32)	3.03 (1.22)	3.82 (1.46)
**Identification**	4.62 (1.05)	4.99 (1.04)	5.47 (.89)
**Entitativity**	3.45 (1.16)	4.68 (1.20)	4.70 (1.00)
**Belonging**	3.93 (1.23)	5.32 (.83)	5.30 (.76)
	**Idea generation task (group level)**		
	**Control**	**Synchrony**	**Complementarity**
	**(n = 10)**	**(n = 10)**	**(n = 11)**
**Fluency (Number of ideas)**	18.55 (3.89)	15.70 (5.11)	19.18 (6.47)
**Number of original ideas**	9.30 (2.74)	6.85 (4.24)	10.36 (5.16)

### Solidarity

A multilevel regression included both contrasts as group-level predictors for individual-level identification with the group. A marginally significant effect of ψ1 was found, indicating that participants who had a coordinated interaction identified more with the group than participants in the control condition, γ = .61, *SE* = .31, *t*(28) = 1.99, *p* = .056. No significant effect of ψ2 on identification was found, γ = .48, *SE* = .35, *t*(28) = 1.39, *p* = .18, although means were somewhat higher in the complementarity than in the synchrony condition.

A similar regression on feelings of belonging revealed that coordinated interaction increased feelings of belonging compared with the control condition, ψ1: γ = 1.38, *SE* = .24, *t*(28) = 5.73, *p* < .001. ψ2 did not significantly affect belonging, γ = -.01, *t* < 1, *ns*.

Moreover, coordinated interaction led to higher perceived entitativity compared with the control condition, ψ1: γ = 1.25, *SE* = .32, *t*(28) = 3.91, *p* = .001. ψ2 did not significantly affect entitativity, γ = .03, *t* < 1, *ns*.

### Personal value to the group

Results showed that participants who had a coordinated interaction (either in synchrony or complementary) reported higher feelings of personal value to the group than participants in the control condition, ψ1: γ = .70, *SE* = .30, *t*(28) = 2.32, *p* = .03. Importantly, ψ2 also significantly affected participants’ sense of personal value, γ = .78, *SE* = .34, *t*(28) = 2.31, *p* = .03, such that participants in the complementarity condition had a higher sense of personal value to the group than participants in the synchrony condition.

### Mediation

As in Study 2, two different mediation analyses were conducted to test the indirect effects of synchrony (vs. control, dummy D1) and complementarity (vs. control, dummy D2) through a sense of personal value on the indicators of solidarity, following the guidelines by Preacher, Zyphur, and Zhang [47] for conducting a 2-1-1 multilevel mediation. As predicted, no evidence for mediation of the synchrony condition effect, via personal value, on identification emerged (γ = 0.66, *SE* = 0.66, *t*(28) = 1.00, *ns*), nor on entitativity (γ = 0.55, *SE* = 0.52, *t*(28) = 1.07, *ns*), nor on belonging (γ = 0.07, *SE* = 1.51, *t*(28) = .04, *ns*). However, the indirect effect of complementarity (D2) via personal value on identification was significant, γ = 2.34, *SE* = 1.06, *t*(28) = 2.20, *p* = .03, 95% CI [.26; 4.42], as was the indirect effect on entitativity, γ = 1.94, *SE* = .80, *t*(28) = 2.44, *p* = .015, 95% CI [.38; 3.49]. No evidence for an indirect effect via personal value on belonging was found, γ = .23, *SE* = 5.34, *t* < 1, *ns*. Mediation could also be tested by including the original contrasts as predictors. The results of this analysis were similar, but we decided to report the dummy-variables here to facilitate interpretation. As hypothesized, in the complementarity condition, but not in the synchrony condition, participants’ sense of personal value to the group predicted identification and the degree to which the group was perceived as an entity.

### Creativity

The videotapes of the group task were coded by two independent coders. They coded for the number of unique ideas that were generated by the group. Afterwards, each idea was coded for originality on a scale from 1 = not original, to 5 = very original. Ideas were unoriginal when they were often mentioned across groups or commonly known. Original ideas were defined as rare, unusual and/or radical ideas [65]. The number of original ideas was defined as the number of ideas that was rated with a 3 or higher on originality [66]. The interrater reliability [67] for the number of ideas was .80, *p* < .001; for the originality of ideas .69, *p* < .001 and for the number of original ideas per group .61, *p* < .001. This can be interpreted as a medium to strong agreement between the raters [68]. The scores of the two raters were averaged before analysis; means are summarized in Table 5.

Because the ideas were generated in groups, the data was analyzed only at the group level. Analyzing these data with overdispersed Poisson regression (as is recommended for skewed count data by [69]) yields similar results. But because in this study the count data were normally distributed around the mean, we report the OLS regression coefficients here. No effect was found for ψ1, suggesting that a coordinated interaction did not increase idea generation, *b* = -1.11, *ns*, nor did it increase the number or original ideas created, *b* = -1.62, *ns*. However, a trend was found on ψ2, suggesting that groups in the complementarity condition generated more ideas than those in the synchrony condition, *b* = 3.48, *SE* = 2.32, *t*(28) = 1.50, *p* = .145, η^2^ = .08. Moreover, groups in the complementarity condition generated a statistically marginally significant higher number of original ideas than those in the synchrony condition, ψ2: *b* = 3.51, *SE* = 1.84, *t*(28) = 1.91, *p* = .066, η^2^ = .12.

### Discussion

Results show that reading a poem in a coordinated way increased group members’ perceptions of entitativity and feelings of belonging, and, with marginal statistical significance, increased their identification with the group compared to participants in a control condition. Complementarity increased group members’ sense of personal value to the group, which in turn predicted their levels of identification and perceptions of entitativity. Participants who read in synchrony, on the other hand, felt equally valuable to the group to those in the control condition. Thus, when participants were allowed to contribute their unique individual lines in the recital of the poem, this not only augmented their sense of personal value to the group, but also increased their sense of solidarity within the group.

We reasoned that the different structure of the groups and the different room for distinct individual contributions could have consequences for the creativity of these groups. The results show that coordinated communication in itself does not increase fluency or originality in an idea generation task. However, a marginally statistically significant trend suggested that the structure of communication does make a difference for subsequent collaboration: Groups in which individuals engaged in complementary action tended to generate a few more ideas, and in particular more original ideas on a subsequent task, than groups which initially spoke in synchrony. This suggests that groups that are structured around the idea that each individual has a unique value to the group, may show an increase in divergent and creative thinking.

## Study 5

The purpose of Study 5 was to devote attention to two additional issues. We examined an alternative explanation for the equal (or in Study 2 somewhat higher) feelings of solidarity in the complementarity condition: Talking sequentially could be less effortful than the synchronous communication. Incidentally, it could also be hypothesized that efforts would be reduced in the synchrony condition, following for instance the literature on social loafing [71]. However, as complementarity represents a more frequently occurring situation (i.e., turn-taking in a conversation), we expected people to be very accustomed to this version of the task, which therefore requires less effort.

Research on fluency has shown that the subjective ease with which people process information influences their judgment on a range of social dimensions (e.g. liking, truthfulness, etc.; see [70] for a review). Extrapolating from this, it is possible that the relative ease of the complementary task in comparison to the synchrony tasks (at least in Study 2 and 4) increased feelings of solidarity in this condition. In Study 5, this alternative explanation was examined by adding a condition in which complementarity was made more effortful. If feelings of solidarity were to be caused by the ease of turn-taking, rather than by the complementary coaction itself as we hypothesized, this should be reflected by higher levels of solidarity in the complementarity normal effort condition, compared to the complementarity high effort condition.

In addition, Study 5 examined whether a sense of personal value was solely important to solidarity because of self-investment, or whether the value of *other* group members would similarly play a role in the development of a sense of solidarity. Conceptually, this would be quite important to know: If the value of *others* were to play a role in emergent sense of solidarity in complementary collaborations, this would be direct evidence that the process of creating solidarity is not entirely self-centered, but that it is a group process, in which contributions of others play a role as well.

### Method

Participants were 150 undergraduate students (*M*age = 19.48, *SD* = 2.41, 75% female) who participated in triads (n = 40) or dyads (n = 15) in a study for partial course credit or a single reward of 6 euros. Groups were randomly assigned to the conditions of a study in which interpersonal coordination was manipulated (synchronous vs. complementarity normal effort vs. complementarity high effort) by reading a poem.

Participants were seated around a table behind individual laptops. After filling out the informed consent form, participants of all groups were instructed to read a fragment of the poem “*Mei*” (Dutch for “May”) by *Herman Gorter*. Participants were instructed to recite the poem from their computer screen. Sentences turned red at the moment they were supposed to be recited by the participant. In the synchrony condition, participants were instructed to recite the poem simultaneously with the other participants, in the same rhythm. In both complementarity conditions, participants were instructed to take turns when reciting the lines of the poem. However, the computer was programmed such that in the complementarity normal effort condition sentences turned red in a rhythm that would allow for smooth transition of speaking turns. However, in the high effort complementarity condition, the sentences turned red in an unpredictable and disordered rhythm. In order to have a coordinated interaction (i.e. without interruptions), participants needed to be alert to changes in rhythm and adjust their speech tempo to the others.

Before starting, participants were given the time to read the poem, then listened to an audiotape of the first two verses of the poem, and finally engaged in a practice session. The practice session involved reciting the first two verses following the instruction for the assigned condition. When the instructions were clear, participants recited the whole poem in the instructed manner. Afterwards, participants completed a questionnaire on their laptops containing measures of entitativity (α = .83), belonging (α = .85), identification (all subscales except for the centrality subscale, α = .93), and sense of personal value to the group (α = .87). Furthermore, we added three rephrased personal value questions to examine the degree to which participants felt that each of the *other* group members was of value to the group (e.g. “*I think the person on my right/left is indispensable to the group*”). Scores correlated highly for both other group members (*r* = .80), and were therefore combined. The total scale of *perceived value of others to the group* had a high reliability (α = .91). To assess the level of effort participant rated their agreement with the statements the task was exacting, easy (reverse coded), required a lot of effort (1 = strongly disagree, 7 = strongly agree). Participants were debriefed and given the opportunity to ask question before leaving the laboratory.

### Results

Again, two orthogonal Helmert contrasts were specified: ψ1 differentiated between the synchrony condition and both complementarity conditions, ψ2 differentiated between the normal effort and the high effort complementarity condition. The ICC1’s for entitativity (.26), belonging (.14), identification (.20) and sense of personal value to the group (.16), and perceived value of others to the group (.13) indicated that multilevel analysis was required. Therefore, data was screened as in Study 4, which led to the removal of one multilevel outlier (Standardized residual on one of the dependent variables > 3). Means are summarized in Table 6.

**Table 6:** Means (SD’s) per condition for the dependent variables in Study 5.

	Synchrony	Complementarity normal effort	Complementarity high effort
	(n = 49)	(n = 50)	(n = 50)
**Personal Value to Group**	2.99 (1.19)	3.91 (1.41)	3.96 (1.45)
**Perceived Value of Others**	3.49 (1.13)	4.27 (1.38)	4.45 (1.26)
**Entitativity**	3.91 (1.14)	4.15 (.80)	4.12 (.99)
**Belonging**	4.30 (1.11)	4.61 (.91)	4.51 (.85)
**Identification**	3.74 (1.04)	3.96 (.73)	3.77 (.81)
**Effort**	3.61 (.99)	3.13 (.99)	3.55 (1.18)

### Manipulation Check

First, we tested whether participants in the high effort complementarity condition would indeed perceive the task to be more effortful than those in the complementarity normal effort condition. This was indeed the case, ψ2: γ = .43 *SE* = .21, *t*(52) = 2.02, *p* = .05. No difference was found in effort between the synchrony and the two complementarity conditions, ψ1: γ = -.27 *SE* = .19, *t*(52) = 1.42, *ns*.

### Solidarity

The regression included both contrasts as group-level predictors for individual-level indicators of solidarity. As expected, we found no differences between the synchrony and the complementarity conditions in levels of identification, ψ1: γ = .05, *t* < 1, *ns*, perceptions of entitativity, ψ1: γ = .07, *t* < 1, *ns*, or feelings of belonging ψ1: γ = .13, *t* < 1, *ns*. Unlike the alternative explanation would suggest, we did not find a difference between the normal effort and high effort complementarity conditions on either identification, ψ2: γ = -.13, *t* < 1, *ns*, entitativity, ψ2: γ = .06, *t* < 1, *ns*, or belonging ψ2: γ = -.01, *t* < 1, *ns*. Thus, the level of effort that was needed to coordinate behavior did not affect levels of identification, perceptions of entitativity or feelings of belonging.

### Value to the group

As predicted, participants who interacted in synchrony reported a lower sense of personal value than participants in both complementarity conditions, ψ1: γ = .87, *SE* = .25, *t*(52) = 3.47, *p* = .001. In addition, ψ2 did not significantly affect feelings of personal value, γ = .12, *t* < 1, *ns*, suggesting that the higher sense of personal value to the group in the complementarity is not explained by the lower levels of effort that the task required.

Similar results were found on the perceived value of the other group members; participants in both complementarity conditions perceived the others to have higher value to the group than participants in the synchrony condition did, ψ1: γ = .81, *SE* = .22, *t*(52) = 3.62, *p* = .001. No differences were found between the participants in the high effort and normal effort complementarity condition, ψ2: γ = 0.23, *t* < 1, *ns*.

### Mediation

We examined whether there was an indirect effect of complementarity (vs. synchrony) via sense of personal value to the group on the indicators of solidarity [47]. To test the complete model, both contrasts were group level predictors in the analysis, personal value was an individual level mediator and entitativity, identification, and belonging were individual level dependent variables. Results showed the predicted effect of ψ1 via sense of personal value on identification, γ = .91, *SE* = .35, *t*(55) = 2.61, *p* = .009, 95% CI [.23; 1.60], and entitativity, γ = 1.19, *SE* = .48, *t*(55) = 2.50, *p* = .012, 95% CI [.26; 2.12], but not on belonging, *t* < 1, *ns*.

Importantly, the effects on entitativity and identification were not only mediated by a sense of personal value to the group, but also by the perception that *others* were valued: Indirect effect on identification, γ = 1.24, *SE* = .35, *t*(55) = 3.53, *p* < .001, 95% CI [.55; 1.94], and entitativity, γ = 1.67, *SE* = .56, *t*(55) = 3.00, *p* = .003, 95% CI [.58; 2.76]. If anything, the mediation by sense of personal value of others appeared to be slightly stronger. In fact, a sense of personal value was highly positively correlated to the experienced value of others (*r* = .75), suggesting that the perceived importance of self positively relates to the perceived importance of others in the group. Again, no mediation was found for the effects on belonging, *t* < 1, *ns*.

### Discussion

The results of Study 5 replicate that an increased sense of personal value in the complementarity conditions compared to the synchrony condition mediate the effects on feelings of identification and perceptions of group entitativity. Thus, when acting complementary, rather than acting in synchrony, a sense of personal value to the group explains the emergence of feelings of solidarity.

Importantly, results show that the extent to which others are valued is just as predictive of the level of solidarity as a sense of own value to the group is. This finding reveals that the forming of solidarity is not primarily self-centered in nature: It is a group process in which contributions of others as well as self play a role. Although asking about the perceived value of others in the group may elicit social desirability concerns, we see no reason why social desirability concerns would play a larger role in one condition than the other. Accordingly, these concerns could not explain why value of others in the group plays a larger role in the development of solidarity in the complementarity condition, than in the development of solidarity in the uniformity condition.

In the complementarity high effort condition, the task was structured in a way that it was difficult to coordinate speech. Note that when designing the experiment, we originally predicted that the varying rhythm of turn-taking would indeed disrupt participants’ ability to successfully take turns. When running the experiment, however, we noticed that participants were able to vary speech rates so fluently that there were very few disruptions: Participants were reluctant to interrupt each other. Instead, they tried to speak faster or stopped their sentence when another participant started speaking. It appeared that the motivation to have a smoothly coordinated interaction was so high that people were able to obtain a smooth flow despite the impediments. We thus conclude that individuals are able to coordinate their actions even if this requires extra effort (see also [72]), and that this ability helps them to acquire feelings of solidarity. Thus, the data of Study 5 provided no support for the alternative explanation that alternating speech would elicit solidarity because it requires less effort than speaking in synchrony.

## Summary of Results across Studies

**Fig 1:**
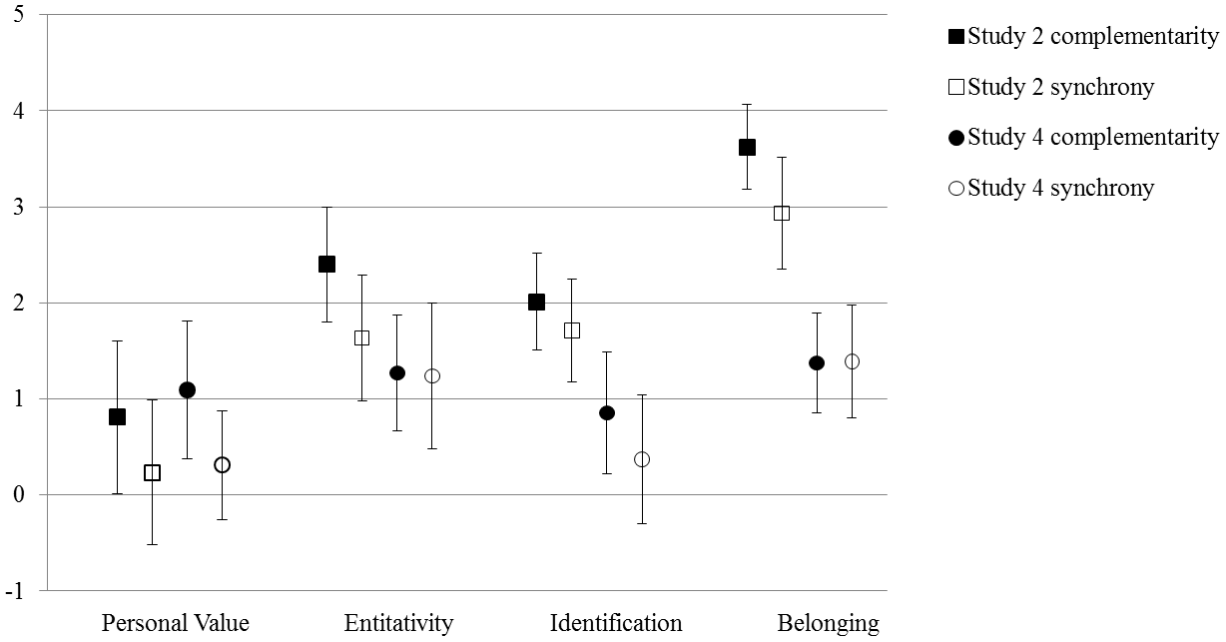
Dummy coded effects (and 95% *CI*s) of synchrony and complementarity (vs. control) for personal value to the group and the three indicators of solidarity.

**Fig 2:**
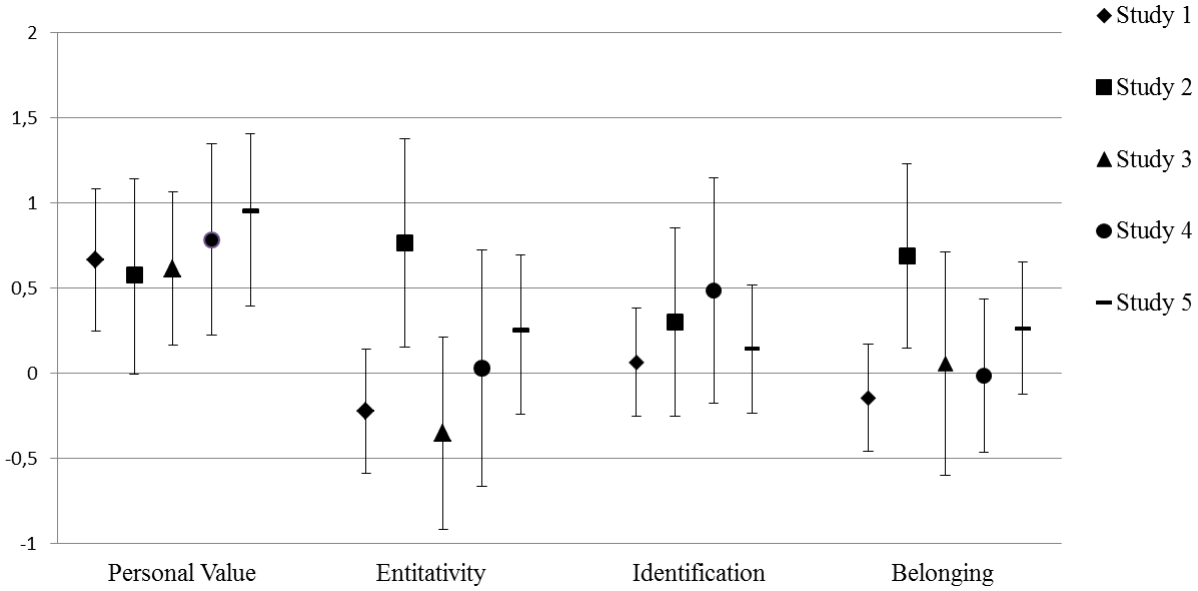
Contrast estimates (and 95% *CI*s) comparing the effects of complementarity and synchrony on personal value to the group and the three indicators of solidarity for Study 1–5.

**Fig 3:**
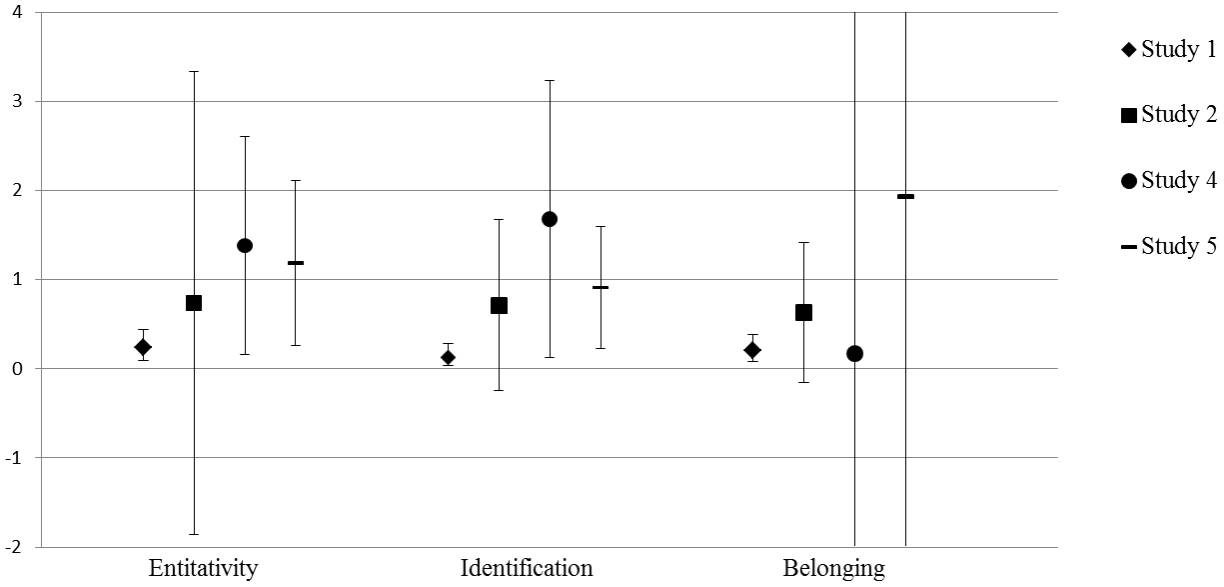
95% confidence intervals of the indirect effects of Contrast 2 (complementarity vs. synchrony) via personal value to the group on the different indicators of solidarity in Study 1, 2, 4, and 5.

Figs 1–3 present a graphical overview of the parameters across the five studies. The hypothesis that both synchronous and complementary action leads to an increased sense of solidarity in comparison with a control condition was tested in Study 2 and Study 4. Initially, Study 3 was also designed to have a control condition: The condition in which participants sang solo. However, singing solo in front of the other group members appeared to be quite a special experience in which processes of solidarity formation also occurred. Moreover, as participants performed their solo parts successively, this condition became somewhat similar to the complementarity condition. In hindsight, we thus believe this condition is not an appropriate control condition, and therefore we should not view comparisons with this condition as convincing evidence for the presence or absence of an increase of solidarity.

In the results section of the individual studies, we used ψ1 to compare both coordinated action conditions jointly to the control condition. Although the positive effects of this contrast indicate that coordinated action serves solidarity, our contrast coding does not allow for the conclusion that each of the conditions differ from control. Fig 1 therefore summarizes the results by providing the parameter estimates and confidence intervals for the *dummy-coded* effects on entitativity, identification, and belonging (thereby comparing synchrony and complementarity separately to the control condition). The hypothesis was generally supported across the two studies: All six confidence intervals for the effect of complementarity on solidarity were higher than zero. Moreover, five out of six confidence intervals on synchrony were well above zero. Moreover, as depicted in Fig 2, no structural differences between the synchrony and complementarity conditions were found with regard to the three indicators of solidarity. Only in Study 2, scores on entitativity and belonging were higher in the complementarity than in the synchrony condition.

Fig 1 also provides support for the second hypothesis; that complementary action increases members’ sense of personal value to the group, whereas synchrony does not. Both Study 2 and Study 4 showed that the confidence intervals for the effect of complementary action on personal value to the group did not include zero, whereas the confidence intervals for the effect of synchrony on personal value to the group did include zero. In line with this, Fig 2 displays contrast estimates comparing the effects of complementary action and synchrony across all five studies. In line with the hypothesis, the 95% confidence interval for the contrast between complementarity and synchrony on personal value does not include zero in any of the studies except Study 2 (95% CI [-.01; 1.16], the smaller effect in Study 2 could be explained by the inclusion of dyads in this study, whereas the other studies mainly included triads—see also the discussion section of Study 2), suggesting that participants experience higher personal value to the group in the complementarity conditions compared to the synchrony conditions.

The final hypothesis concerns the indirect effect of personal value. We expected that the increased sense of personal value to the group in the complementarity condition compared to the synchrony condition indirectly explains the experience of solidarity. This hypothesis was tested in all studies except Study 3, due to the complex nature of the design. In Study 1, 4, and 5, we found support for an indirect effect of complementary action (vs. synchrony) via personal value to the group on perceptions of group entitativity and identification with the group, as none of the 95% confidence intervals for the indirect effect included zero (see Fig 3). In Study 2, the indirect effects were in the same direction, but the confidence intervals did include zero (CI entitativity [-1.86;3.34], CI identification [-.25;1.68]). On the third indicator of solidarity—feelings of belonging—results were mixed: Although the results for belonging in Study 1 and 2 were broadly similar to the results for entitativity and identification, in Study 4 and 5 the confidence intervals for feelings of belonging were very large and included zero (CI belonging Study 4 [-7.40; 7.73], CI belonging Study 5 [-13.65; 17.51]).

Finally, the studies had too little power to reliably compare the correlations within conditions. Possibly as a result, these correlations did not show a very clear pattern. We compared the relationships between indispensability and each of the indicators of solidarity in both the uniformity and the complementarity conditions. Correlations ranged between .07 and .50, and no significant between-condition differences emerged (all *Z*s < 1.19, *p*s > .23). Thus, although we found a general positive relation between feeling personally valuable to the group and experiencing solidarity, we found no evidence that this relation was stronger in the complementarity condition than in the uniformity condition. However, we note that due to power constraints, one should be cautious in interpreting differences in the magnitude of correlations within conditions.

## General Discussion

The present research shows that during coordinated action, processes of identity formation take place. Findings suggest that solidarity can emerge as a result of different forms of coordinated action: Uniform action, in which similarities between group members are central and individuality is in the background; and more complementary forms of action, in which the individual actions of each group member contributes to the emergence of solidarity. To differentiate these processes of group formation, we identify sense of personal value to the group as a mediator. More specifically, the current studies reveal that compared to people who act in uniform ways (e.g. synchronously), people who act in ways complementary to each other have a higher sense of personal value to the group (Studies 1, 3, 4 and 5), which relates to an increased level of identification and perception of group entitativity (Studies 1, 2, 4 and 5). These findings contribute to the literature in a number of ways.

First, the results suggest that identity formation can occur as a side effect of co-action. Previous research on social identity formation [32–33] has distinguished between deductive processes of identity formation on the one hand, in which groups form their identity by contrasting their own group with relevant outgroups (e.g. [12], [17]) and inductive processes on the other hand, in which a group is based on the individual contributions of its members. It has been suggested that the route through which solidarity emerges defines the nature of the group: Whereas deductively formed groups allow for little variation between individuals within the group, inductively formed groups can be strengthened by individual differences of their members [35].

The present research extends this research. In particular it sheds light on processes of induction, by showing that the way in which individuals coordinate their actions influences the nature of the solidarity. But although the results for complementary action are directly relevant to inductive social identity formation, we point out that the synchrony findings are not directly attributable to deductive social identity formation. The reason is that although synchrony relies on the process of deduction, it may do so in the absence of a shared social identity derived from superordinate commonalities (cf. [32–33]). Indeed, although in our experiments group actions were coordinated through experimental instructions, none of our studies ensured that a shared social identity was made salient. Although there are situations in which synchrony is predefined by a higher order that could be construed as a shared identity (e.g., in the army, or in a directed orchestra), synchrony is often defined by the entrainment of the behavior between different individuals (e.g. [6], [72]). Thus, the proper conclusion from the present research, we believe, is that synchronous action in groups creates a sense of solidarity in which individuals feel connected at an overarching level of ‘we’, in which individual contributions are of secondary importance. Moreover, synchronous action may create a group structure in which individual distinctiveness is problematic and therefore leaves less room for creativity.

Second, the present research identifies a sense of personal value to the group as a mediator of these effects. More specifically, findings show that when individuals behave in a complementary way, for instance when performing a group task in which they have distinguishable contributions, or when having a conversation in which they take turns, a sense of solidarity is developed on the basis of members’ feelings of being an essential component of the group. In contrast, in groups that are structured by similarity, like a choir singing in unison or an army in which soldiers march synchronously, a sense of personal value to the group does not play such a critical role in the process of identification. Our results show that complementary and synchronous co-action are equally likely to increase solidarity within the group, but differ in whether they position the individual in the foreground, or in the background of group formation.

These results provide insight in the role of individuality in groups. Although the need to belong to groups and the need for personal distinctiveness may sometimes be contrasting needs (e.g., [73]), the present research illustrates that in certain settings this need not be the case. Our results show that accentuating individual contributions in a group may promote, rather than reduce identification with a group, as this underlines the value of individuals to the group. This finding is in line with research which shows that in inductively formed groups, member heterogeneity may contribute to identification processes [2]. We extend this finding by showing that in addition to groups that are formed in an inductive way, coordinated action of a complementary nature can similarly underline the essentiality of distinct individual contributions to the group. In addition, the present research identifies the critical role of a sense of personal value to the group in identification processes.

Third, the results of Study 4 suggest that groups with complementary structures may be more successful when generating ideas in subsequent collaboration tasks. More specifically, a trend was found which suggested that complementary action groups generated more, and more creative, ideas than groups that had previously acted in synchrony. Although this finding should be interpreted with caution—the effects were only marginally significant and based on a relatively small number of groups—we believe that it provides a potential direction for future research. More specifically, it points to the possibility that compared to groups which acquire solidarity through acting uniformly, complementary groups may be more likely to think divergently; which has been argued to contribute to creativity, problem solving and decision making [54], [63]. Future research could further examine whether different forms of co-action can improve group performance on for instance creative or decision-making tasks.

Finally, in Study 5 we show that although in complementary groups the focus is more on the individual, this should not be equated with self-centeredness. Instead, Study 5 reveals that perceiving other members as valuable is at least as important in predicting identification and entitativity, as is the sense of personal value to the group. It thus appears that in complementarity groups, it is not only critical that one is ‘being heard’. Instead, it is the combination of individual inputs from self and others that predicts feelings of solidarity. Indeed, Study 3 shows that singing solo in a choir increases ones sense of *voice*—or the feeling that one is being heard. However, this did not result in increased perceptions of entitativity. In contrast, the subjective feelings of value of self and others *both* relate to perceptions of the group as an entitative whole, suggesting that self and others are treated as similarly important not just in groups founded upon uniformity, but also in groups founded upon complementary actions. This is a conclusion with important implications, for it implies that group systems that are founded upon complementarity need not be intrinsically more competitive or more prone to inequality. But since the conclusion is based on results of a single study, we emphasize that this would be an important issue for future research.

The five studies conducted in this research used different methods to test the proposed model. Findings were replicated in several contexts, making use of naturally occurring groups in an online study (Study 1), and manipulated groups in controlled lab environments (Studies 2 & 5) and field studies (Studies 3 & 4) with different samples from the general population, undergraduate students, singers, and actors respectively. The coordination activities that were examined included activities performed in naturally occurring groups, such as sports, talking, making assignments, organizing events etc. (Study 1), the act of singing together (Study 3), reciting stories via headsets (Study 2) or reciting poems in either a free rhythm (Study 4) or a directed rhythm (Study 5). By exploring different methods we may have sacrificed some experimental control, which could have affected the tightness of our results. However, we believe that testing our model in different contexts increased the ecological validity of our findings.

## Limitations and Directions for Future Research

One important caveat is that (in the nature of experimental research) we attempted to differentiate idealized states in which group solidarity either emerges from uniform vs. complementary action. Of course, this notion of two types of processes is likely to present an overly simplistic view on reality. We believe that most groups rely on both complementary and uniform inputs from its members, and therefore both processes described here should be evident, to a greater or lesser extent, in all groups in society. Nevertheless, the results of Study 1 do suggest that it may be fruitful to make this distinction even in real-life groups.

Another potential limitation of the current research is that the manipulations to elicit synchronous or complementary action in Studies 2–5 implicitly direct towards a common goal: The completion of the story, poem, or song. Consequently, the effects of coordinating group members’ actions may partly result from cooperatively working towards a goal, rather than of the coordinated interaction per se. This indicates that we should be careful generalizing our findings to forms of coordinated interaction that occur within a less clear task structure. There are however two reasons to believe that the results do not occur as a function of task structure alone. First, research on complementary and synchronous rituals in communities without a clearly defined task structure (Buddist chanting, Brazilian drumming) showed increased entitativity compared to control groups in which rituals were performed without synchrony or complementarity [23]. Second, the identification of personal value to the group as a mediator for the effects of complementary (compared to synchronous) action suggest that these different forms of interaction elicit *qualitatively different* forms of solidarity.

One more minor issue concerns some slight variations in findings across studies. First it is important to point out where there was no variability: We found relatively similar results across all indicators of solidarity, with coordinated action increasing feelings of belonging, levels of identification, and perceptions of entitativity. Although we had no a priori expectations for differences between these three constructs, the literature does suggest that they are distinct indicators that capture different aspects of solidarity. Whereas entitativity is defined as the overarching sense of unity that group members experience, identification is concerned with the relation of the individual with the group. Previous research suggests that these constructs are closely related (e.g., [2], [74]), and also in our studies we generally find high correlations (see Table 2). Moreover, in our studies, we confirmed that the effects on perceived entitativity and identification were both mediated by a sense of personal value to the group.

But effects on belongingness were slightly more elusive: Although effects on belongingness were broadly consistent, in Study 4 and 5 no mediation was found. Although it is difficult to interpret null effects (especially across studies with only modest power) we may speculate that part of the reason for this could lie with the measure used: The Need Threat Scale [42]. In this scale, items of belonging are mixed with items of rejection, such as the reversed item “I felt accepted by the others group members”. We successfully used this as a measure of solidarity in a previous line of research, in which belonging was threatened by a disruption of conversational coordination [9–10], [30]. However, in the current studies no such threat is present: Participants can coordinate successfully in both the synchrony and the complementarity condition—they just use different ways of coordination. Perhaps this absence of any threat may explain why this scale proved to be less sensitive in the present studies.

## Conclusions

In the present research we show that a sense of ‘us’ can emerge in the background of specific actions that individuals perform together, but that the nature of these actions (complementary or synchronous) shapes the groups via different pathways. This sense of ‘us’ consists not just of perceptions of group entitativity but also a sense of individual identification to the group. This confirms that dynamic processes in small groups can take on a more categorical and more interactive shape, both of which produce a sense of solidarity. The crucial difference between these two processes is not the level of solidarity they produce, but its quality: Categorical processes relegate individual group members to the background of group formation. In interactive processes, by contrast, individuals are at the forefront of what it means to be “us”.

## References

[1] Postmes T, Spears R, Lee T, Novak R (2005) Individuality and social influence in groups: Inductive and deductive routes to group identity. J Pers Soc Psychol 89: 747–763. 16351366 10.1037/0022-3514.89.5.747

[2] Jans L, Postmes T, Van der Zee KI (2011) The induction of shared identity: The positive role of individual distinctiveness for groups. Person Soc Psychol Bull 37: 1130–1141. 10.1177/0146167211407342 21525328

[3] Lickel B, Hamilton DL, Wieczorkowska G, Lewis A, Sherman SJ, Uhles AN (2000) Varieties of groups and the perception of group entitativity. J Pers Soc Psychol 78: 223–246. 10707331 10.1037//0022-3514.78.2.223

[4] Dasgupta N, Banaji MR, Abelson RP (1999) Group entitativity and group perception: Associations between physical features and psychological judgment. J Pers Soc Psychol 77: 991–1003. 10573876 10.1037//0022-3514.77.5.991

[5] Lakens D (2010) Movement synchrony and perceived entitativity. J Exp Soc Psychol 46: 701–708.

[6] Marsh KL, Richardson MJ, Schmidt RC (2009) Social connection through joint action and interpersonal coordination. Top Cogn Sci 1: 320–339. 10.1111/j.1756-8765.2009.01022.x 25164936

[7] McGarty C, Haslam SA, Hutchinson KJ, Grace DM (1995) Determinants of perceived consistency: The relationship between group entitativity and the meaningfulness of categories. Br J Soc Psychol 34: 237–256. 7551771 10.1111/j.2044-8309.1995.tb01061.x

[8] Gaertner L, Schopler J (1998) Perceived ingroup entitativity and intergroup bias: An interconnection of self and others. Eur J Social Psychol 28: 963–980.

[9] Koudenburg N, Postmes T, Gordijn EH (2013) Conversational flow promotes solidarity. PLoS ONE 8: e78363 10.1371/journal.pone.0078363 24265683 PMC3827030

[10] Koudenburg N, Postmes T, Gordijn EH (2014) Conversational flow and entitativity: The role of status. Br J Soc Psychol 53: 350–366. 10.1111/bjso.12027 23431961

[11] Campbell DT (1958) Common fate, similarity, and other indices of the status of aggregates of persons as social entities. Behav Sci 3: 14–25.

[12] Turner JC (1985) Social categorisation and the self-concept: A social cognitive theory of group behaviour In: Lawler E. J., editor. Advances in group processes: Theory and research Vol 2 Greenwich, CT: JAI Press pp. 77–122.

[13] Durkheim E (1984) The division of labor in society. London: Macmillan. Original work published in 1893.

[14] Leach C, van Zomeren M, Zebel S, Vliek M, Pennekamp S, Doosje B, et al (2008) Group-level self-definition and self-investment: A hierarchical (multicomponent) model of in-group identification. J Pers Soc Psychol 95: 144–165. 10.1037/0022-3514.95.1.144 18605857

[15] Byrne D, Griffitt W (1969) Similarity and awareness of similarity of personality characteristic determinants of attraction. J Exp Res Pers 3: 179–186.

[16] Byrne D, Griffitt W, Stefaniak D (1967) Attraction and similarity of personality characteristics. J Pers Soc Psychol 5: 82–90. 4382219 10.1037/h0021198

[17] Turner JC (1982) Towards a cognitive redefinition of the group In: Tajfel H., editor. Social identity and intergroup relations. Cambridge: Cambridge University Press pp. 15–40.

[18] Turner JC, Hogg MA, Oakes PJ, Reicher SD, Wetherell MS (1987) Rediscovering the social group: A self-categorization theory. Oxford: Blackwell.

[19] Hogg MA, Turner JC (1987) Intergroup behaviour, self‐stereotyping and the salience of social categories. Br J Soc Psychol 26: 325–340.

[20] Bernieri FJ, Rosenthal R (1991) Interpersonal coordination: Behavior matching and interactional synchrony In: Feldman RS, Rime B, editors. Fundamentals of nonverbal behavior: Studies in emotion & social interaction. New York: Cambridge University Press pp. 401–432.

[21] Cappella JN (1996) Dynamic coordination of vocal and kinesic behavior in dyadic interaction: Methods problems, and interpersonal outcomes In: Watt JH, VanLear CA, editors. Dynamic patterns in communication processes. pp. 353–386.

[22] LaFrance M (1982) Posture mirroring and rapport: Interaction rhythms. New York: Human Sciences Press.

[23] Fischer R, Callander R, Reddish P, Bulbulia J (2013) How do rituals affect cooperation? Hum Nat 24: 115–125. 10.1007/s12110-013-9167-y 23666518

[24] Hove MJ, Risen JL (2009) It's all in the timing: Interpersonal synchrony increases affiliation. Soc Cogn 27: 949–960.

[25] Valdesolo P, DeSteno D (2011) Synchrony and the social tuning of compassion. Emotion 11: 262–266. 10.1037/a0021302 21500895

[26] Miles LK, Nind LK, Henderson Z, Macrae CN (2010) Moving memories: Behavioral synchrony and memory for self and others. J Exp Soc Psychol 46: 457–460.

[27] Paladino MP, Mazzurega M, Pavani F, Schubert TW (2010) Synchronous multisensory stimulation blurs self-other boundaries. Psychol Sci 21: 1202–1207. 10.1177/0956797610379234 20679523

[28] Wiltermuth SS (2012) Synchronous activity boosts compliance with requests to aggress. J Exp Soc Psychol 48: 453–456.

[29] Gaertner L, Iuzzini J, Guerrero Witt M., Oriña MM (2006) Us without them: Evidence for an intragroup origin of positive ingroup regard. J Pers Soc Psychol 90: 426–439. 16594829 10.1037/0022-3514.90.3.426

[30] Koudenburg N, Postmes T, Gordijn EH. (2011) Disrupting the flow: How brief silences in group conversations affect social needs. J Exp Soc Psychol 47: 512–515.

[31] Prentice DA, Miller D, Lightdale JR (1994) Asymmetries in attachment to groups and to their members: Distinguishing between common-identity and common-bond groups. Pers Soc Psychol Bull 20: 484–493.

[32] Postmes T, Haslam SA, Swaab RI (2005) Social influence in small groups: An interactive model of social identity formation. Eur Rev Social Psychol 16: 1–42.

[33] Postmes T, Spears R, Cihangir S (2001) Quality of decision making and group norms. J Pers Soc Psychol 80: 918–930. 11414374

[34] Swaab R, Postmes T, van Beest I, Spears R (2007) Shared cognition as a product of, and precursor to shared identity in negotiations. Pers Soc Psychol Bull 33: 187–199. 17259580 10.1177/0146167206294788

[35] Jans L, Postmes T, Van der Zee KI (2012) Sharing differences: The inductive route to social identity formation. J Exp Soc Psychol 48: 1145–1149.

[36] Clark HH (1996). Using Language. Cambridge: Cambridge University Press.

[37] Gambi C, Pickering MJ (2011). A cognitive architecture for the coordination of utterances. Front. Psychol 2: 275 10.3389/fpsyg.2011.00275 22065961 PMC3206582

[38] Wilson M, Wilson TP (2005) An oscillator model of the timing of turn-taking. Psychon Bull Rev 12: 957–968. 16615316 10.3758/bf03206432

[39] Bettencourt B, Sheldon K (2001) Social roles as mechanism for psychological need satisfaction within social groups. *J Pers Soc Psychol* 81: 1131 11761313

[40] Stryker S (1987) Identity theory: developments and extensions. Oxford: John Wiley & Sons pp 332.

[41] Vignoles VL, Chryssochoou X, Breakwell GM (2000) The distinctiveness principle: Identity meaning, and the bounds of cultural relativity. Pers Soc Psychol Rev 4: 337–354.

[42] Van Beest I., Williams KD (2006) When inclusion costs and ostracism pays. ostracism still hurts. J Exp Soc Psychol 91: 918–928. 10.1037/0022-3514.91.5.918 17059310

[43] Hayes AF (2012) PROCESS: A versatile computational tool for observed variable mediation, moderation, and conditional process modeling [white paper]. Available: http://www.afhayes.com/public/process2012.pdf. Accessed 6 August 2018.

[44] Preacher KJ, Kelley K (2011) Effect size measures for mediation models: Quantitative strategies for communicating indirect effects. Psychol Methods 16: 93–115. 10.1037/a0022658 21500915

[45] Simmons JP, Nelson LD, Simonsohn U (2011) False-positive psychology undisclosed flexibility in data collection and analysis allows presenting anything as significant. Psychol Sci 22: 1359–1366. 10.1177/0956797611417632 22006061

[46] Bliese P (2000) Within-group agreement, non-independence, and reliability: Implications for data aggregation and analysis In: Klein KJ, Kozlowski SWJ, editors. Multilevel theory, research, and methods in organizations: Foundations extensions, and new directions. San Francisco: Jossey-Bass pp. 349–381.

[47] Preacher KJ, Zyphur MJ, Zhang Z (2010) A general multilevel SEM framework for assessing multilevel mediation. Psychol Methods 15: 209–233. 10.1037/a0020141 20822249

[48] Durkheim E (1954) The elementary forms of religious life. London: Allen & Unwin. Original work published 1912.

[49] Anderson B (1991) Imagined communities: Reflections on the origins and spread of nationalism. London: Verso.

[50] Bellah RN (2006) Durkheim and ritual In: Bellah RN, Tipton SM, editors. The Robert Bellah reader. London: Duke University Press pp. 150–80.

[51] Müller V, Lindenberger U (2011). Cardiac and respiratory patterns synchronize between persons during choir singing. *PloS one* 6: e24893 10.1371/journal.pone.0024893 21957466 PMC3177845

[52] Festinger L, Thibaut J (1951) Interpersonal communication in small groups. J Abnorm Soc Psychol 46: 92–99. 10.1037/h0054899 14813892

[53] Marques JM, Paez D (1994) The ‘black sheep effect’: Social categorization, rejection of ingroup deviates, and perception of group variability. Eur Rev Social Psychol 5: 37–68.

[54] Nemeth CJ (1986) The differential contributions of majority and minority influence. Psychol Rev 93: 23–32.

[55] Nemeth CJ, Kwan J (1987) Minority influence, divergent thinking and the detection of correct solutions. J Appl Soc Psychol 17: 786–797.

[56] Stasser G, Taylor LA, Hanna C (1989) Information sampling in structured and unstructured discussions of three- and six-person groups. J Pers Soc Psychol 57: 67–78.

[57] Stasser G (1988) Computer simulation as a research tool: The DISCUSS model of group decision making. J Exp Soc Psychol 24: 393–422.

[58] Stasser G, Stewart DD, Wittenbaum GM (1995) Expert roles and information exchange during discussion: The importance of knowing who knows what. J Exp Soc Psychol 31: 244–265.

[59] Hornsey MJ, Jetten J, McAuliffe BJ, Hogg MA (2006) The impact of individualist and collectivist group norms on evaluations of dissenting group members. J Exp Soc Psychol 42: 57–68.

[60] Moscovici S, Lage E (1976) Studies in social influence III: Majority versus minority influence in a group. Eur J Social Psychol 6: 149–174.

[61] Hoffman LR, Maier NRF (1961) Quality and acceptance of problem solutions by members of homogeneous and heterogeneous groups. J Abnorm Soc Psychol 62: 401–407. 13715029 10.1037/h0044025

[62] Mannix E, Neale MA (2005) What differences make a difference? the promise and reality of diverse teams in organizations. Psychol Sci Public Interest 6: 31–55. 26158478 10.1111/j.1529-1006.2005.00022.x

[63] Guilford JP (1956) The structure of intellect. Psychol Bull 33: 267–293. 10.1037/h0040755 13336196

[64] Back KW (1988) Metaphors for public opinion in literature. Public Opin Q 52: 278–288.

[65] Rietzschel EF, Nijstad BA, Stroebe W (2007) Relative accessibility of domain knowledge and creativity: The effects of knowledge activation on the quantity and originality of generated ideas. J Exp Soc Psychol 43: 933–946.

[66] Paulus PB, Kohn NW, Arditti LE (2011) Effects of quantity and quality instructions on brainstorming. J Creat Behav 45: 38–46.

[67] McGraw KO, Wong SP (1996) Forming inferences about some intraclass correlation coefficients. Psychol Methods 1: 30–46.

[68] LeBreton JM, Senter JL (2008) Answers to 20 questions about interrater reliability and interrater agreement. Organ Res Methods 11: 815–852.

[69] Coxe S, West SG, Aiken LS (2009) The Analysis of Count Data: A Gentle Introduction to Poisson Regression and Its Alternatives, J Pers Assessment, 91: 121–136, 10.1080/00223890802634175 19205933

[70] Alter AL, Oppenheimer DM (2009) Uniting the tribes of fluency to form a metacognitive nation. Pers Soc Psychol Rev 13: 219–235 10.1177/1088868309341564 19638628

[71] Karau SJ, Williams KD (1993) Social loafing: A meta-analytic review and theoretical integration. J Pers Soc Psychol 65.

[72] Richardson MJ, Marsh KL, Isenhower R, Goodman J, Schmidt RC (2008) Rocking together: dynamics of intentional and unintentional interpersonal coordination. Hum Mov Sci 26: 867–891. 10.1016/j.humov.2007.07.002 17765345

[73] Brewer M (1991) The social self: On being the same and different at the same time. Person Soc Psychol Bull 17: 475–482.

[74] Castano E (2004) On the advantages of reifying the ingroup In: Yzerbyt V, Judd CM, Corneille O, editors. The psychology of group perception: Perceived variability, entitativity, and essentialism. New York, NY: Psychology Press pp. 381–400.

